# Suspicious cold thyroid nodule with intense focal 68Ga-DOTATATE uptake: a case report

**DOI:** 10.1186/s41824-022-00126-5

**Published:** 2022-04-19

**Authors:** Ringo Manta, Wendy Delbart, Ivan Duran Derijckere, Marie Quiriny, Pieter Demetter, Patrick Flamen, Ioannis Karfis

**Affiliations:** 1 Nuclear Medicine Department, Institut Jules Bordet, Université Libre de Bruxelles (ULB), Brussels, Belgium; 2 Surgical Oncology Department, Institut Jules Bordet, Université Libre de Bruxelles (ULB), Brussels, Belgium; 3 Pathology Department, Institut Jules Bordet, Université Libre de Bruxelles (ULB), Brussels, Belgium

**Keywords:** Thyroid nodule, Somatostatin receptor, ^68^Ga-DOTATATE PET/CT

## Abstract

A 51-year-old male was found with bilateral thyroid nodules on ultrasonography neck imaging. The largest nodule, measuring 23 × 26 × 35 mm, was located in the left lobe and was classified as EU-TIRADS 4. Thyroid function tests were normal, as were serum levels of parathormone, Chromogranin A, carcinoembryonic antigen and calcitonin. The nodule was cold on thyroid scintigraphy. Fine-needle aspiration of the nodule did not demonstrate cellular atypia. High focal uptake was found on both ^111^In-DTPA-octreotide scintigraphy and ^68^Ga-DOTATATE PET/CT. Histopathological analysis showed a microfollicular adenoma without malignancy. Immunohistochemical staining did not suggest neuroendocrine neoplasia or C cell hyperplasia. However, high expression of somatostatin receptor 2 (SSTR2) was observed in the microfollicular adenoma compared to the surrounding healthy tissue, with predominant localization in the endothelial cells and at the secretory pole of the thyroid epithelial cells in contact with blood vessels. High focal thyroid uptake on ^68^Ga-DOTATATE PET/CT can be observed in benign thyroid nodules due to an overexpression of SSTR by endothelial cells. However, incidental focal thyroid uptake on SSTR imaging requires further investigations to rule out thyroid malignancy.

## Introduction

Somatostatin receptor (SSTR) imaging methods, such as ^111^In-DTPA-octreotide scintigraphy and ^68^Ga-DOTATATE PET/CT, are mainly indicated for the workup of neuroendocrine tumors (NET) (Özgüven et al. 2021). As this imaging modality is becoming increasingly used, incidental focal uptake in benign tissues or organs has been described (e.g., inflammatory lymph nodes, pituitary adenoma and meningioma). We present a case of a patient with intense and focal SSR expression in a cold thyroid nodule.

## Case report

**Fig. 1.**
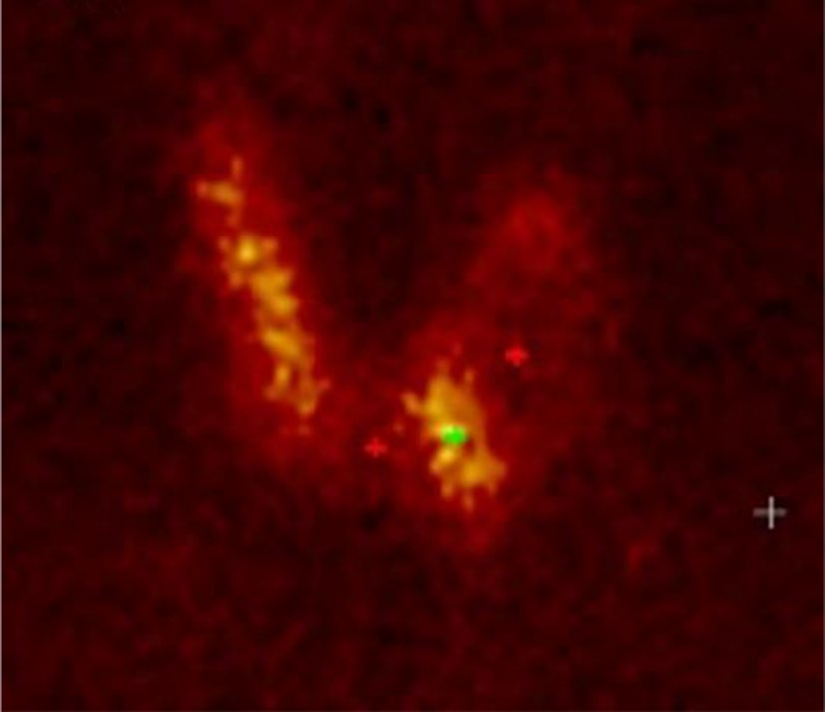
^99m^Tc planar thyroid scan showing a cold left thyroid nodule

**Fig. 2.**
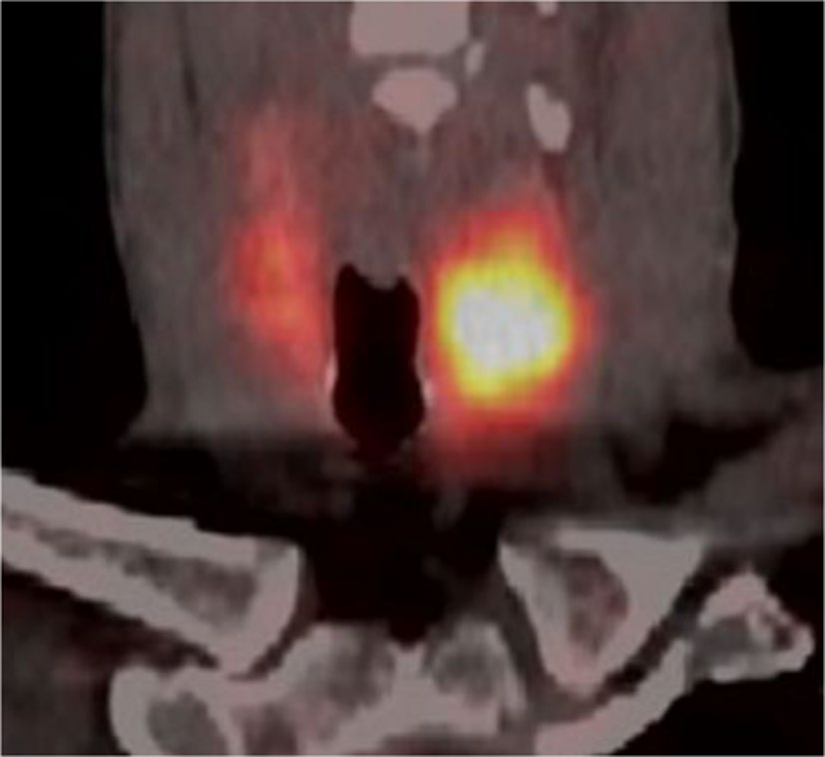
^68^Ga-DOTATATE PET/CT coronal fusion image showing high uptake in the left thyroid nodule and mild and diffuse physiological uptake in the right lobe

**Fig. 3:**
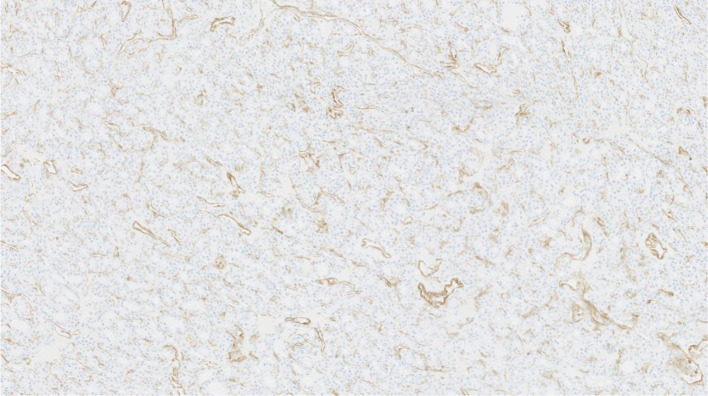
SSTR2 immunohistochemistry staining in the adenomatous nodule showing prominent immunostaining in the endothelial cells

**Fig. 4:**
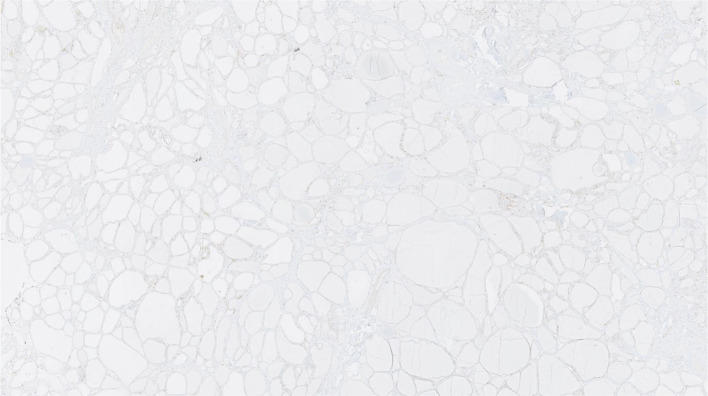
SSTR2 IHC staining of healthy surrounding thyroid tissue

A 51-year-old male presented with significant weight loss and fatigue for several months with a history of hypertension and gastroesophageal reflux disease. Clinical examination revealed a left palpable thyroid nodule. All routine blood tests, including a complete hemogram, renal function and liver function, were normal. Thyroid function tests were within the normal range as were serum levels of parathormone (PTH), Chromogranin A (CgA), carcinoembryonic antigen (CEA) and calcitonin. Ultrasonographic neck imaging showed bilateral intermediate-risk EU-TIRADS 4 thyroid nodules. The largest nodule, measuring 23 × 26 × 35 mm, was located in the left lobe. Thyroid scintigraphy using technetium-99 m pertechnetate showed a cold nodule located in the left lobe (Fig. 1). Fine-needle aspiration biopsy (FNAB) of the nodule did not demonstrate cellular atypia (Bethesda category II). For unknown reasons, ^111^In-DTPA-octreotide scintigraphy was requested by the treating physician and displayed a high focal uptake in the left nodule. The same findings were observed on the ^68^Ga-DOTATATE PET/CT, with a SUV_max_ of 13.8 (Fig. 2). No other sites of pathological uptake were detected. The patient was discussed at the multidisciplinary tumor board meeting. Given the presence of bilateral thyroid nodular lesions, the decision was to perform a near-total thyroidectomy associated with a prophylactic neck dissection of level VI. Histopathological findings revealed a multinodular goiter containing a microfollicular adenoma without malignancy. Immunohistochemical analysis of calcitonin, CgA, synaptophysin and somatostatin receptor subtype 2 (SSTR2) expression was performed. Results did not suggest neuroendocrine neoplasia or C cell hyperplasia. Higher expression of SSTR2 was observed in the microfollicular adenoma compared to the surrounding healthy tissue, with predominant localization in the endothelial cells and at the secretory pole of the thyroid epithelial cells in contact with blood vessels (Figs. 3, 4).

## Discussion

The prevalence of thyroid nodules in the general adult population is up to 60%. Thyroid malignancy accounts for approximately 1–10% of incidental thyroid nodules (Fisher and Perrier 2018; Durante et al. 2018). Ultrasonography allows the assessment of various morphological features suggesting malignancy. European Thyroid Imaging and Reporting Data System (EU-TIRADS) features include irregular margins, non-oval shape, microcalcifications and hypoechogenicity (Russ et al. 2017). Thyroid scintigraphy is used to determine the functional status of a nodule as non-functioning nodules are associated with a 10 to 20% risk of malignancy, while most hyperfunctioning nodules are benign (Maia and Zantut-Wittmann 2012). Both morphological and functional features help to select nodules for US-guided FNAB. However, results are inaccurate in approximately 10 to 30% of cases (Maia and Zantut-Wittmann 2012).

SSTR are highly expressed at the surface of neuroendocrine cells and can be found in many tissues, including normal thyroid tissue (Özgüven et al. 2021). Beyond expression in NETs, expression of SSTR was also demonstrated in other tumors such as lymphoma, small cell lung carcinoma and prostate cancer or benign lesions such as sarcoid granulomas (Banerjee and Pomper 2013). SSTR expression can be detected by scintigraphy and PET/CT using radiolabeled somatostatin analogues such as 111In-octreotide and 68Ga-DOTATATE, respectively.

Increased thyroid uptake on SSTR imaging has been described in various thyroid disorders such as Hashimoto's thyroiditis, papillary thyroid cancer and medullary thyroid cancer (Lincke et al. 2009). In a retrospective study, thyroid uptake was evaluated in 237 patients who underwent ^68^Ga-DOTATATE PET/CT to localize unknown primary and metastatic neuroendocrine tumors (Nockel et al. 2016). Among fourteen patients with incidental focal thyroid uptake, three (21%) had papillary thyroid cancer. No malignancy was detected among the remaining eleven patients, including six patients (42%) with adenomatous nodules and one patient (7%) with lymphocytic thyroiditis.

In the present case, the incidental finding of a high focal thyroid uptake with a SUV_max_ of 13.8 as detected by ^68^Ga-DOTATATE PET/CT raised suspicion for thyroid malignancy. Histopathological and immunohistochemical analyses revealed a benign adenomatous nodule with overexpression of SSTR2. Remarkably, the high density of SSTR2 was predominantly localized in endothelial cells. High endothelial expression of SSTR has been reported in both malignant and benign lesions such as lymphoma, hemangioma and thymoma (Ruuska et al. 2018; Brogsitter et al. 2014; Ferone et al. 2001). The presence of SSTR on endothelial cells is thought to contribute to tissue growth, however, precise clinical significance remains unclear (Watson et al. 2001).

## Conclusion

High focal thyroid uptake on ^68^Ga-DOTATATE PET/CT can be observed in benign thyroid nodules due to an expression of SSTR by endothelial cells. However, incidental focal thyroid uptake on SSTR imaging requires further investigations to rule out thyroid malignancy.

## Data Availability

Not applicable.
